# A Simple Predictive Score to Distinguish between Disseminated Histoplasmosis and Tuberculosis in Patients with HIV

**DOI:** 10.3390/jof8010016

**Published:** 2021-12-27

**Authors:** Mathieu Nacher, Kinan Drak Alsibai, Loïc Epelboin, Philippe Abboud, Frédégonde About, Magalie Demar, Félix Djossou, Romain Blaizot, Maylis Douine, Nadia Sabbah, Nicolas Vignier, Leila Adriouch, Aude Lucarelli, Mathilde Boutrou, Pierre Couppié, Antoine Adenis

**Affiliations:** 1 CIC INSERM 1424, Centre Hospitalier de Cayenne, 97300 Cayenne, French Guiana; maylis.douine@ch-cayenne.fr (M.D.); nicolas.vignier@ch-cayenne.fr (N.V.); antoine.adenis@ch-cayenne.fr (A.A.); 2 Département Formation Recherche, Université de Guyane, 97300 Cayenne, French Guiana; pierre.couppie@ch-cayenne.fr; 3 Service d’anatomopathologie, Centre Hospitalier de Cayenne, 97300 Cayenne, French Guiana; kdrak@doctor.com; 4 Service des Maladies Infectieuses et Tropicales, Centre Hospitalier de Cayenne, 97300 Cayenne, French Guiana; loic.epelboin@ch-cayenne.fr (L.E.); philippe.abboud@ch-cayenne.fr (P.A.); fredegonde.about@ch-cayenne.fr (F.A.); felix.djossou@ch-cayenne.fr (F.D.); mathilde.boutrou@ch-cayenne.fr (M.B.); 5 Laboratoire Polyvalent, Centre Hospitalier de Cayenne, 97300 Cayenne, French Guiana; magalie.demar@ch-cayenne.fr; 6 Unité Mixte de Recherche Tropical Biome and Immunopathology, Université de Guyane, 97300 Cayenne, French Guiana; 7 Service de Dermatologie, Centre Hospitalier de Cayenne, 97300 Cayenne, French Guiana; romain.blaizot@ch-cayenne.fr; 8 Service de Médecine, Centre Hospitalier de Cayenne, 97300 Cayenne, French Guiana; nadia.sabbah@ch-cayenne.fr; 9 Coordination Régionale de la Lutte Contre le VIH et les Hépatites, Centre Hospitalier de Cayenne, 97300 Cayenne, French Guiana; leila.adriouch@ch-cayenne.fr (L.A.); aude.lucarelli@ch-cayenne.fr (A.L.)

**Keywords:** disseminated histoplasmosis, tuberculosis, advanced HIV, predictive score

## Abstract

Disseminated histoplasmosis is a common differential diagnosis of tuberculosis in disease-endemic areas. We aimed to find a predictive score to orient clinicians towards disseminated histoplasmosis or tuberculosis when facing a non-specific infectious syndrome in patients with advanced HIV disease. We reanalyzed data from a retrospective study in Cayenne Hospital between January 1997–December 2008 comparing disseminated histoplasmosis and tuberculosis: 100 confirmed disseminated histoplasmosis cases and 88 confirmed tuberculosis cases were included. A simple logit regression model was constructed to predict whether a case was tuberculosis or disseminated histoplasmosis. From this model, a score may be obtained, where the natural logarithm of the probability of disseminated histoplasmosis/tuberculosis = +3.917962 × WHO performance score (1 if >2, 0 if ≤2) −1.624642 × Pulmonary presentation (1 yes, 0 no) +2.245819 × Adenopathies > 2 cm (1 yes, 0 no) −0.015898 × CD4 count − 0.001851 × ASAT − 0.000871 × Neutrophil count − 0.000018 × Platelet count + 6.053793. The area under the curve was 98.55%. The sensitivity of the model to distinguish between disseminated histoplasmosis and tuberculosis was 95% (95% CI = 88.7–98.3%), and the specificity was 93% (95% CI = 85.7.3–97.4%). In conclusion, we here present a clinical-biological predictive score, using simple variables available on admission, that seemed to perform very well to discriminate disseminated histoplasmosis from tuberculosis in French Guiana in well characterized patients.

## 1. Introduction

With an HIV prevalence greater than 1% for over 3 decades, French Guiana is the French territory where the human immunodeficiency virus (HIV) epidemic is most preoccupying [1]. Disseminated histoplasmosis and tuberculosis have consistently remained among the top AIDS-defining illnesses, disseminated histoplasmosis being the first [2,3]. During HIV infection, both histoplasmosis and tuberculosis are often disseminated infections. In persons with advanced HIV, in the absence of treatment, the dissemination of the pathogen may cause a rapid and potentially fatal evolution, often in a context of hemophagocytic lymphohistiocytosis [4,5]. In the absence of rapid diagnostic tests, invasive diagnostic methods are often necessary and presumptive treatment is often given guided by both knowledge of the local epidemiology—the respective incidences of disseminated histoplasmosis and tuberculosis—and clinical judgement [6]. Although the clinical dilemma is often framed as a dichotomy between disseminated histoplasmosis or tuberculosis, in fact, coinfections are common [7]. The biological confirmation through pathogen identification by culture is long and may be difficult, although direct examination or pathology may yield rapid results [8]. Although progress is in the pipeline [9,10,11], in practice, rapid and sensitive antigenic detection techniques are still not available in most endemic countries [12,13]. The non-specific nature of the clinical and paraclinical findings for both diseases makes the differential diagnosis between disseminated histoplasmosis and tuberculosis difficult in disease-endemic areas [14]. Ever since the first publication by Samuel Darling [15], numerous publications have reported cases of histoplasmosis resembling tuberculosis. We have recently mapped estimates for histoplasmosis and tuberculosis incidence and case fatality for Latin America showing that, for a median scenario of 50% of symptomatic histoplasmosis cases and a historical level of 40% case fatality, 9/21 (43%) Latin American countries had an equivalent of greater incidence of disseminated histoplasmosis than tuberculosis, and that 14/21 (67%) countries had an equivalent or greater number of disseminated histoplasmosis deaths than tuberculosis [16]. 

In French Guiana, because of a good knowledge of the epidemiologic context, clinicians generally suspect histoplasmosis and tuberculosis in immunosuppressed patients at admission [4,17]. Although lengthy hospitalizations are often required before the pathogen is identified, despite proactive sampling of fluids and tissues, presumptive treatment is common in order to avoid potentially fatal therapeutic delays, usually antituberculosis therapy prevailing on antifungal therapy [18,19]. Although it has long been assumed that these two diseases are similar, this was not based on any direct comparison. In this context, we had performed a comparative study between tuberculosis and histoplasmosis, suggesting that, although there were many similarities, there were some differences that allowed experienced physicians to distinguish between the two diagnoses [14]. Hence, tuberculosis was more associated with pulmonary signs and an elevated CRP, whereas histoplasmosis was associated with cytopenia and/or a digestive presentation. Whether these striking features at the level of a study population had any value when caring for an individual patient was not clear. Since then, to our knowledge, no other direct comparisons have been made. We aimed to further the analyses to determine if we could find a predictive score to orient clinicians towards disseminated histoplasmosis or tuberculosis when facing a non-specific infectious syndrome in patients with advanced HIV disease.

## 2. Materials and Methods

### 2.1. Study Design

A retrospective study took place at Cayenne Hospital (Cayenne, French Guiana) between January 1997–December 2008 [14]. The study population consisted of patients from the HIV hospital cohort, which is part of the French Hospital Database on HIV, for which data have been systematically collected since 1992.

### 2.2. Inclusion and Exclusion Criteria

The inclusion criteria were an age ≥ 18 years, hospital admission or outpatient visit before admission, inclusion in the French Hospital Database, confirmed HIV infection, confirmed tuberculosis by culture and identification of *Mycobacterium tuberculosis* or confirmed disseminated histoplasmosis by direct examination and/or culture of *Histoplasma capsulatum*, and biological screening less than 7 days before treatment initiation. The inclusion date was that of treatment initiation for tuberculosis or disseminated histoplasmosis.

The exclusion criteria were concomitant tuberculosis and histoplasmosis, a history of tuberculosis or histoplasmosis, and an immune reconstitution disease due to tuberculosis or disseminated histoplasmosis. If the diagnosis of tuberculosis or histoplasmosis was only done by polymerase chain reaction, we did not include the patient.

Clinical evaluation of the patient’s general condition upon admission used the Eastern World Health Organization performance status score. Statistical analysis of anonymized was performed with Stata 16 (Stata Corporation, College Station, TX, USA). A pulmonary presentation was defined as clinical symptoms or signs of the bronchopulmonary sphere (cough, dyspnea, abnormal auscultation) or abnormal chest X-ray. This was coded as a dichotomous variable. Clinically palpable lymphadenopathies >2 cm were also coded as a dichotomous variable.

Based on our previous analysis and stepwise multivariate model [14], we selected variables to construct multiple logistic regression models. The dependent variable was tuberculosis or disseminated histoplasmosis, respectively, coded 0 or 1. Hence, if the OR was significantly < 1, the variable was associated with tuberculosis, and, if the OR was significantly > 1, then the variable was associated with histoplasmosis. Biological variables—CD4, Neutrophils, Aspartate-Amino-Transferase (ASAT), and Platelets—were included as continuous variables in order to make marginal predictions for different values of these variables. We did not include “local” variables, such as the place of residence or the duration of stay in French Guiana or ethnicity, because these variables would not be transposable in other contexts. We used Akaike’s Information Criterion to select the most parsimonious model, and we used Hosmer-Lemeshow’s goodness of fit test. We then used the logit coefficients to construct a predictive score. We performed postestimation analyses estimating sensitivity, specificity, and positive and negative predictive values. Finally, we plotted the full model’s ROC curve. The alpha risk was set at 5%. For continuous variables, the reference laboratory threshold used 1 for those with platelets < 150,000/mm^3^, and 0 for those with higher platelet counts. For CD4 and for neutrophil counts, we used the median value as a cutoff, 1 being lower that the median, and 0 being higher that the median. To avoid overfitting, we also tried the model on a training random sample of the dataset and then used the coefficient from the training model to make predictions on the testing set with the remaining observations. To compute the probability of having histoplasmosis, we exponentiated the logit coefficient from the training model and then divided the odds by the odds + 1 − Probability = odds/(1 + odds).

### 2.3. Ethical and Regulatory Aspects

The study and database were approved by the Institut National de la Santé et de la Recherche Médicale (INSERM (IRB00000388, FWA00005831). Patients gave written informed consent for the study and the publication of results.

## 3. Results

Table 1 shows the most parsimonious Logit regression model obtained on 188 observations, 100 confirmed disseminated histoplasmosis cases, and 88 confirmed tuberculosis cases. From this model, a score may be obtained, where the natural logarithm of the probability of histoplasmosis compared to tuberculosis = +3.917962 × WHO performance score (1 if >2, 0 if ≤2) −1.624642 × pulmonary presentation (1 yes, 0 no) +2.245819 × adenopathies > 2 cm (1 yes, 0 no) −0.015898 × CD4 count − 0.001851*ASAT − 0.000871 × neutrophil count − 0.000018 × platelet count + 6.053793. Supplementary file 1 provides an Excel spreadsheet computing the probability for the specific values of an individual patient.

**Figure 1:**
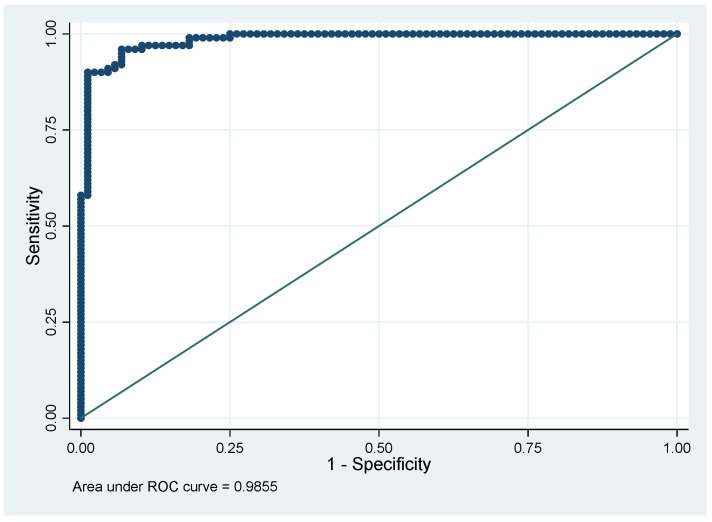
The performance of the model with an area under the ROC curve of 98.55%.

**Figure 2:**
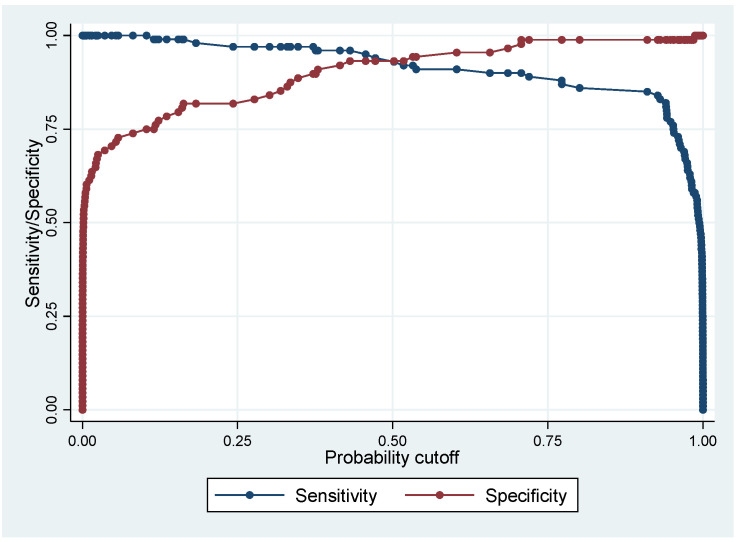
The evolution of sensitivity and specificity for different cutoffs with an optimal cutoff value of about 0.5.

Figure 1 shows the ROC curve of the predictive model and Figure 2 shows the evolution of sensitivity and specificity for different cutoffs.

Table 2 shows the performance of the multivariate model in classifying disseminated histoplasmosis and tuberculosis. Sensitivity of the model to distinguish between disseminated histoplasmosis and tuberculosis was 95% (95% CI = 88.7–98.3%), and the specificity was 93% (95% CI = 85.7.3–97.4%). The positive predictive value was 93.9%, and the negative predictive value was 93%, but the respective numbers did not correspond to real life proportions. Overall, 94.1% of patients were correctly classified.

We looked at the patients that were wrongly classified (data not shown). For missed disseminated histoplasmosis, there were too few misclassified observations to do robust statistics, but the missed disseminated histoplasmoses tended to have higher mean platelet counts than correct predictions, 27,0142 versus 165,032 per mm^3^, respectively, and higher mean neutrophil counts, 2838 versus 2276 per mm^3^, respectively. To eliminate overfitting artefacts, we randomly split the dataset into a training set and a testing set, where the coefficients obtained from the training set (*n* = 94) were used to compute the probability on the testing set (*n* = 94). With this, the sensitivity on the training set was 83% (70–91%), and specificity was 91.8% (80–97.7%).

**Figure 3:**
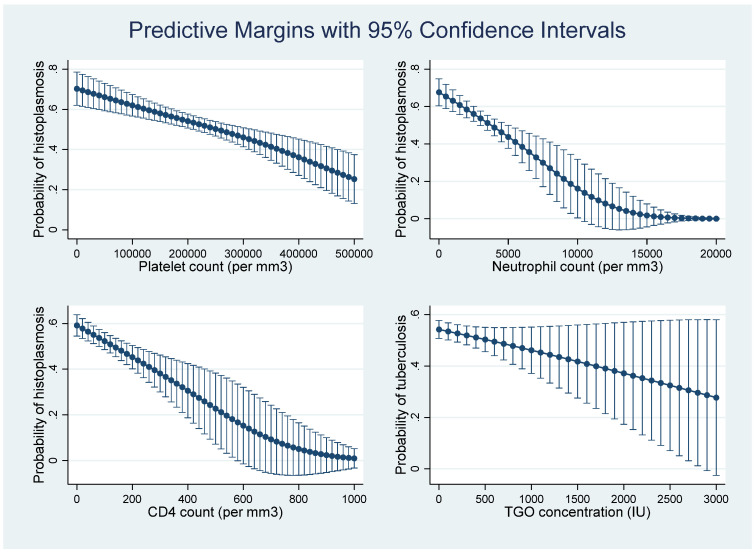
The evolution of the probability of having disseminated histoplasmosis, rather than tuberculosis, for CD4, neutrophil, and platelet counts, and Aspartate-Amino-Transferase concentration.

Figure 3 showed that, according to the multivariate model and the predictive score derived from it, as platelet counts and neutrophil counts decline, and as CD4 counts decline, the probability of disseminated histoplasmosis increases. This was not clear for Aspartate-Amino-Transferase concentrations.

## 4. Discussion

Here, we show that, when comparing microbiologically confirmed cases of disseminated histoplasmosis and tuberculosis, a model using variables that are available in any hospital was able to correctly identify cases of tuberculosis or disseminated histoplasmosis with great accuracy, as shown by an area under the ROC curve of 98.55%. Although some authors have tried to discriminate patients [20], this is the first study to calculate sensitivity and specificity of variables to distinguish disseminated histoplasmosis from tuberculosis. The wide availability of variables from common clinical and paraclinical data could make it very useful for clinicians who wish to distinguish between the two in endemic contexts, a situation that corresponds to much of Latin America [16].

In French Guiana, disseminated histoplasmosis has been high on the research and clinical agendas, and microbiological facilities are well developed, but we have observed that patients on antituberculosis drugs with no confirmed diagnosis had a twofold increased risk of dying than those with a positive diagnosis [21]. Unfortunately, antigen detections tests or urine LAM for tuberculosis have still not been implemented as routine in patients with advanced HIV disease. Thus, the present score may be of future use to avoid misdiagnoses and treatment delays.

The limitations of the present study are that, although this comparison models a common and emblematic differential diagnosis, the back-to-back comparison of disseminated histoplasmosis and tuberculosis is reductive and falsely dichotomizes real clinical situations with advanced HIV, where other differential diagnoses may be evoked. The diagnosis of disseminated histoplasmosis and tuberculosis relied on microbiology, the gold standard, but we now know that the sensitivity of these methods is lower than other methods of antigen testing for disseminated histoplasmosis or Gene Xpert for tuberculosis [22]. Therefore, perhaps there are a number of more difficult diagnoses that are not captured by this model. Furthermore, for the sake of clarity, we excluded coinfections with both tuberculosis and disseminated histoplasmosis, a situation that is not uncommon and may even be very frequent in some epidemiological contexts [7]. Superficial lymphadenopathies were clinically measured, which may have introduced some variability and imprecision. The relatively small sample size could be seen as a weakness, but the magnitude of the area under the curve, and the fact that training and testing models on randomly split samples yielded similar results, suggests that overfitting was not a problem. As the context is specific to French Guiana, the score extracted should nevertheless not be taken at face value and replace sound clinical practice until it has been validated elsewhere in different contexts, a task that seems feasible given the near universal availability of the variables composing the score. Furthermore, as other diagnostic methods are scaled up across Latin American hospitals, and beyond, the score may require further validation and recalibration. Positive and negative predictive values are probably not valid because the relative proportion of disseminated histoplasmosis and tuberculosis in our sample did not necessarily reflect the real situation. Further prospective studies could help obtain these values for the site where the study is performed.

## 5. Conclusions

In conclusion, we here present a clinical-biological predictive score, using simple variables available on admission, that seemed to perform very well to discriminate disseminated histoplasmosis from tuberculosis in French Guiana in well characterized HIV patients. This should be confirmed by external validation in different epidemiological contexts, and beyond the dichotomy disseminated histoplasmosis-tuberculosis, before we see if it is useful for clinicians who most often still do not have access to rapid antigen detection tests, and for patients for whom therapeutic delays can lead to early death. Although Samuel Darling’s differential diagnosis remains common at the bedside, this data suggests that, in fact, in patients with advanced HIV, single infections by *M. tuberculosis* or *H. capsulatum* are quite different.

## Figures and Tables

**Table 1:** Logit regression of the most parsimonious model according to Akaike’s information criterion.

Disseminated Histoplasmosis vs. Tuberculosis	Coef.	St. Err.	*p*-Value	[95% Conf Interval]	
WHO performance score > 2	3.917962	0.806778	0.000	2.337	5.499
Pulmonary presentation	−1.624642	0.891495	0.068	−3.372	0.123
Adenopathies > 2 cm	2.245819	0.907807	0.013	0.467	4.025
CD4 count (per mm^3^)	−0.015898	0.004648	0.001	−0.025	−0.007
ASAT (IU)	−0.001851	0.000899	0.039	−0.004	−0.000
Neutrophil count (per mm^3^)	−0.000871	0.000240	0.000	−0.001	−0.000
Platelet count (per mm^3^)	−0.000018	0.000004	0.000	−0.000	−0.000
Intercept	6.053793	1.755852	0.001	2.612	9.495

**Table 2:** Performance of the multivariate model to classify patients as disseminated histoplasmosis or tuberculosis.

	Confirmed Disseminated Histoplasmosis	Confirmed Tuberculosis	Total
Classified as disseminated histoplasmosis	95	6	101
Classified as tuberculosis	5	82	87
Total	100	88	188

## Data Availability

Anonymized data can be made upon reasonable request at cicec@ch-cayenne.fr.

## Supplementary Materials

The following are available online at https://www.mdpi.com/article/10.3390/jof8010016/s1, Supplementary file 1: Probability calculator to distinguish histoplasmosis from tuberculosis.
